# Relationships Between Smartphone Dependency and Aggression Among Middle School Students: Mediating and Moderating Effects of Ego-Resilience, Parenting Behaviour, and Peer Attachment

**DOI:** 10.3390/ijerph16193534

**Published:** 2019-09-21

**Authors:** Youn-Joo Um, Yun-Jung Choi, So Yeon Yoo

**Affiliations:** 1 Red Cross College of Nursing, Chung-Ang University, Seoul 06974, Korea; 2002242@naver.com; 2 Department of Nursing, Kyungil University, Gyeongbuk 38428, Korea

**Keywords:** smartphone, dependency, aggression, ego-resilience, parenting behaviour, peer attachment

## Abstract

This study examined the moderating and mediating effects of ego-resilience, parenting behaviour, and peer attachment on the relationship between smartphone dependency and aggression. Participants included 1863 middle school youths who used smartphones and had responded to the 7th Korean Children and Youth Panel Survey (KCYPS), which was conducted by Korea’s National Youth Policy Institute. Data were analysed using descriptive statistics, correlation, and hierarchical regression analyses. First, ego-resilience showed a partial mediating effect on the relationship between smartphone dependency and aggression (significant moderating effects were revealed). Second, parenting behaviour demonstrated a partial mediating effect on the relationship between smartphone dependency and aggression (no moderating effects observed). Third, peer attachment had a partial mediating effect on the relationship between smartphone dependency and aggression (no moderating effects were observed). These findings suggest that mental health and student growth can be improved as children develop by implementing various nursing and health care programs designed to improve ego-resilience, parenting behaviour, and peer attachment.

## 1. Introduction

Developments in digital media technology have resulted in substantial changes in everyday life. The Information and Communication Policy Institute indicates that usage rates among adolescents have rapidly increased, from 5.9% in 2010 to 40% in 2011 and 92% in 2017 [1]. The risk group exhibiting a strong dependency on smartphones among middle school students was determined at 30.4%; this is relatively high compared to the adult addiction rate of 17.4%. Smartphone usage time was also the highest among middle school students [2]. 

Smartphone dependency is significantly higher than other lifestyles due to excessive smartphone use (salience), the inability to personally control smartphone use (self-control failure), and physical, psychological, and social negative consequences (serious consequences) due to smartphone use. Nevertheless, smartphone dependency refers to the continuous use of smartphones [3]. While smartphones have various functions and additional applications (apps) that can be used, participants reported that the more gaming entertainment they participated in, the more pictures they took, the more frequent their social media usage and texts, and the more frequently they watched videos, the higher their dependency on smartphones became. On the other hand, if they frequently talked to their families or listened to music, they reported less dependency on their smartphones [4].

Brenner [5] noted that the addictive use of media causes younger users to experience related problems at an exponential rate. Middle school students tend to be especially sensitive and vulnerable to small problems, such as a lack of self-restraint and the consideration of others. These individuals are most likely to develop aggressive behaviours that will persist throughout their lives [6]. Smartphone usage is also more likely to cause school violence by inhibiting sociality and causing students to lose their ability to cope with emotional stress or peer conflict due to their exposure to violent and suggestive applications [7]. School violence between peers has recently become the most prevalent social problem resulting from aggression [8]. This increase in aggression needs to be fully confronted since it may attract physical and verbal violence among peer groups. Such escalations also increase the rate at which children imitate adult crimes and the possibility that major social problems will occur in adulthood [9].

There is a statistically significant and positive correlation between smartphone dependency and aggression [10]. Lakens [11] reported that smartphone overuse among middle and high school students may cause difficulty in controlling anger. Ko, et al. [12] reported that smartphone dependency negatively affected emotional and behavioural issues among adolescents, including conduct disorder. 

Previous research in this area has primarily focused on the biological, social, and emotional characteristics of smartphone-addicted users [13]. However, findings have provided insufficient information about how to prevent smartphone dependency that leads to social problems such as aggression. Individuals, households, and society itself should be concerned with the moderating and mediating effects that smartphones have on student aggression, especially when combined with the effects of the parenting environment (parenting behaviour) and society (peer attachment) on individual character (ego-resilience) [14]. As such, this study examined the reasons for choosing ego-resilience, parental attitudes, and peer behaviour as mediating and moderating variables.

First, ego-resilience is a concept that includes control, positivity, and sociality; it is a psychological construct that individuals use to adapt without showing behavioural or emotional problems resulting from stress, adversity, and/or threatening environments [15].

Ego-resilience was chosen as a mediating variable in this study because many previous studies have used it to determine the causes of internal psychological problems that arise when moving from maladjustment to adaptive situations [6]. Many studies have also been conducted on whether the personal psychological factor of ego-resilience affects smartphone dependency [16,17], but little research has examined how smartphone dependency affects individual adaptability and ego-resilience. 

This study also examined why ego-resilience works as a moderating variable in this context. Previous studies have used ego-resilience as a moderating variable to examine negative situations or behaviours in addition to how it assists with lifestyle adaptation [18]. Recent findings indicate that ego-resilience has a positive influence on school-life adaptation, family relationships, stress, and overuse of the Internet and smartphones [19,20]. 

Previous studies have restricted their mediated approaches to one variable by evaluating only one mediating or moderating effect. However, this study used ego-resilience to comprehensively evaluate the mediated and moderated effects when looking at both the causative and regulating relationships.

Parenting behaviour is an important factor in determining the basic behavioural patterns that a child will develop in the family system while becoming a social being [21]. Research has indicated that parenting behaviour has a mediating effect that is influenced by the causes and consequences of various problematic behaviours [22]. In this context, increased smartphone use among children is now causing a variety of problems between users and parents and is expected to affect the overall parent-child relationship [23]. 

Parenting behaviour has also been shown to have a moderating effect that can strengthen or weaken problematic behaviour among adolescents [24]. Lee’s [25] study on Internet gaming addiction and aggression indicated that parenting behaviour was controlled by interacting with self-restraint and physical symptoms. Further, parental warmth and surveillance had a controlled effect on stress and psychological coordination [22,26]. Thus, the psychological mechanisms by which parenting behaviours affect smartphone dependency can comprehensively be studied by simultaneously analysing the mediating and moderating effects.

Peer relationships are highly important for adolescents who spend substantial amounts of time at school [27]. It is necessary to examine the relationship between smartphone dependency and peer attachment because smartphones are tools that allow adolescents to communicate with their peers regardless of time and place. This study analysed the mediating effects of peer attachment because previous studies on the causal relationship between ‘peer attachment’ and ‘smartphone dependency’ have shown inconsistent and mixed results. Several studies have indicated that increased peer attachment among adolescents is associated with an increased reliance on smartphones; these individuals react sensitively to maintaining peer communication and social relationships in addition to the related smartphone functions [28]. On the other hand, some studies have shown that increased smartphone dependency increases peer attachment aimed at addressing psychological desires; these individuals do not want to be excluded from peer relationships while using smartphones [29]. However, smartphone usage cannot ultimately work as a positive alternative to strengthening trust among peers or preventing exclusion [29]. It is therefore necessary to specifically verify how smartphone dependency affects peer attachment for adolescents. We also analysed peer attachment as a moderating variable because we expected that it would affect the relationship between smartphones and aggression. This assumption was based on previous research showing that adolescent adaptability can be adjusted according to the level of peer interaction. Adolescents with high peer attachment and affinity do not rely on smartphones to conduct real-life conversations [19,28]. Analyses on the effects of peer attachment in adolescent relationships indicate that smartphone dependency and aggression can play an understated role in making subjects more vulnerable to vulnerable situations or, conversely, can reduce aggression. This is critical to prevent adolescent problems. It is thus important to validate both the mediating and moderating effects of peer attachment on smartphone dependency and aggression. Such an analysis can provide basic data on adolescent smartphone dependency.

Following previous studies, this research examined the impact of smartphone dependency among middle school students and tested both the mediating and moderating effects of related individual (ego-resilience), family (parenting behaviour), and social (peer attachment) aspects. We thought it was important to analyse each of these three concepts because individuals should be approached differently in the family context depending on whether there are mediating or moderating effects. 

This study was conducted to identify the impact of smartphone dependency on aggression in addition to the mediating and moderating factors to obtain basic data for use in developing nursing and family counselling programs. Ultimately, this information can be used to improve mental health among adolescents so that they may develop into healthy adults. This study’s hypotheses were as follows:

**Hypothesis** **1.** *Ego-resilience has a mediating effect on the relationship between adolescent smartphone dependency and aggression.*

**Hypothesis** **2.** *Parenting behaviour has a mediating effect on the relationship between adolescent smartphone dependency and aggression.*

**Hypothesis** **3.** *Peer attachment has a mediating effect on the relationship between adolescent smartphone dependency and aggression.*

**Hypothesis** **4.** *Ego-resilience has a moderating effect on the relationship between adolescent smartphone dependency and aggression.*

**Hypothesis** **5.** *Parenting behaviour has a moderating effect on the relationship between adolescent smartphone dependency and aggression.*

**Hypothesis** **6.** *Peer attachment has a mediating effect on the relationship between adolescent smartphone dependency and aggression.*

## 2. Methods

### 2.1. Participants

This was a secondary study that utilised data from middle school freshman panels taken from the 7th Korean Children and Youth Panel Survey (KCYPS), which was conducted by Korea’s National Youth Policy Institute [2]. (Ethical approved project identification code is 1041078-201903-HRSB-099-01). In 2010, the number of samples surveyed by region was allocated in proportion to the number of students in the first and fourth grades of elementary school and the first grade of middle school across 16 cities and provinces. School samples were designed to survey one student class per school; the first survey (in 2010) was conducted through the school, while the 2011 survey was conducted by visiting target households. The seventh survey was conducted from October to December, 2016. Consent was obtained from both the parents and their adolescent children by visiting each household. This information was coded without any personally identifiable information (i.e., anonymised).

Of the 2351 middle school freshmen surveyed, 1863 smartphone users were analysed after excluding respondents that had missing values (see Table 1 for information on age and sex). Adolescent respondents answered questions by writing directly onto the questionnaire. A total of 13 students (0.7%) were aged 14 (born in 2002), while 1799 (96.5%) were aged 13 (born in 2003), and 51 (2.8%) were aged 12 (born in 2004). Of the 1863 analysed students, there were 916 boys (49.2%) and 947 girls (50.8%).

### 2.2. Instruments

#### 2.2.1. Smartphone Dependency

We measured smartphone dependency using the related scale presented in questionnaires distributed by Korea’s National Youth Policy Institute [2]. The National Youth Policy Institute reported that the questionnaire developed by Lee, Kim, and Na [30] was used. The following items were included: ‘I have felt anxious over the past year when not taking a cell phone with me’ and ‘I have felt isolated over the past year when I am without a cell phone’. A total of seven questions were responded to on a four-point Likert scale ranging from ‘very strongly’ (one point) to ’not at all’ (four points); all were reverse-coded. We then determined the sum of all the items for each respondent (Appendix A). Higher scores indicated higher smartphone dependency. Cronbach’s alpha revealed an internal consistency of 0.854.

#### 2.2.2. Aggression

We measured aggression according to the Aggression Scale presented in a questionnaire distributed by Korea’s National Youth Policy Institute [2]. The National Youth Policy Institute reported that the questionnaire developed by Jo and Im [31] and modified by the National Youth Policy Institute was used. Questions related to aggression included the following: ‘Even small mistakes made by others have easily upset me over the past year’ and ‘I have wanted to interfere with what someone else was doing over the past year’. A total of six questions were answered according to a four-point Likert scale ranging from ‘very strongly’ (one point) to ‘not at all’ (four points); all were reverse-coded. We then determined the sum of all the items for each respondent. Higher scores indicated higher aggression. Cronbach’s alpha revealed an internal consistency of 0.809.

#### 2.2.3. Ego-Resilience

We measured ego-resilience using the ego-resilience scale presented in a questionnaire distributed by Korea’s National Youth Policy Institute [2]. The National Youth Policy Institute reported that the questionnaire developed by Block and Kremen [32] and translated by Yoo and Shim [33] and modified by Kwon [34] was used. Items included the following: ‘Even when unexpected things happen, I have been able to get over them quickly over the past year’ and ‘I have been generous with my friends over the past year’. A total of fourteen questions were answered according to a four-point Likert scale ranging from ‘very strongly’ (one point) to ‘not at all’ (four points); all were reverse-coded. We then determined the sum of all the items for each participant. Higher scores indicated higher ego-resilience. Cronbach’s alpha revealed an internal consistency score of 0.831. 

#### 2.2.4. Parenting Behaviour

We measured parenting behaviour related to smartphone dependency using the parenting behaviour scale presented in a questionnaire distributed by Korea’s National Youth Policy Institute [2]. The National Youth Policy Institute reported that the questionnaire developed by Huh [35] and modified by the National Youth Policy Institute was used. Items included the following: ‘My parents have respected my opinions over the past year’, ‘My parents have continually emphasised that I should win when I do things over the past year’, and ‘I have wished my parents were less concerned about me over the past year’. A total of twenty-one items were categorised into the following six subscales: monitoring (three items), affection (four items), reasoning (three items), inconsistency (three items), over-expectation (four items), and intrusiveness (four items). This arrangement was used to distinguish between positive and negative parenting behaviours. Positive parenting behaviours included monitoring, affection, and reasoning. In this study, positive parenting behaviour is generally used to describe situations when parents expressed love to their children, communicated well, and offered reasonable explanations and reasons for discipline. Negative parenting behaviours included inconsistency, over-expectation, and intrusiveness. Further, negative parenting behaviours may have also suggested that parents had too much anxiety about their children, required undesirable things, and/or confused their children through inconsistent behaviour. Items were answered according to a four-point Likert scale ranging from ‘very strongly’ (one point) to ‘not at all’ (four points); positive parenting behaviour items were reverse coded. We then determined the sum of all the items for each participant. Higher scores indicated more positive parenting behaviour, while lower scores indicated more negative behaviour. Cronbach’s alpha indicated an internal consistency of 0.868. 

#### 2.2.5. Peer Attachment 

We measured peer attachment according to the peer attachment scale presented in a questionnaire distributed by Korea’s National Youth Policy Institute [29]. The National Youth Policy Institute reported that the questionnaire developed by Armsden and Greenberg [36] and modified by the National Youth Policy Institute was used. The scale included the following items: ‘My friends have respected my ideas during our conversations over the past year’ and ‘I have felt alone or lonely even when with my friends over the past year’. Items were answered according to a four-point Likert scale ranging from ‘very strongly’ (one point) to ‘not at all’ (four points); all but the three questions on alienation were reverse-coded. We then determined the sum of all the items for each participant. Higher scores indicated greater peer attachment. Cronbach’s alpha revealed an internal consistency of 0.823.

### 2.3. Data Analysis

All data from this study were first used to calculate descriptive statistics for each variable. The reliability of each measuring tool was verified using Cronbach’s α, while Pearson’s correlation was calculated to determine the measured variables. Baron and Kenny [37] conducted a hierarchical regression to determine whether ego-resilience, parenting behaviour, and peer attachment served as mediators between smartphone dependency and aggression. Here, a bootstrapping test using the SPSS PROCESS macro (SPSS Inc., Chicago, IL, USA) was conducted to test the statistical significance of the mediated effects. Finally, a hierarchical regression was performed to test the moderating effects of the ego-resilience, parenting behaviour, and peer attachment variables to determine whether smartphone dependency affected aggression.

## 3. Results

### 3.1. Descriptive Statistics

Table 2 shows the results of calculating score ranges, minimum values, maximum values, means, and standard deviations to examine the general trends of smartphone dependency, aggression, ego-resilience, parenting behaviours, and peer attachment. This study was conducted among 1863 students who used smartphones during the first grade of middle school and responded to the questionnaire.

### 3.2. Correlations Between Major Variables

Smartphone dependency showed a significant correlation with all variables (Table 3). Specifically, smartphone dependency was positively correlated with aggression (r = 0.27, *p* < 0.01), but negatively correlated with ego-resilience (r = −0.27, *p* < 0.01), parenting behaviour (r = −0.25, *p* < 0.01), and peer attachment (r = −0.12, *p* < 0.01). Aggression showed markedly negative correlations with ego-resilience (r = −0.23, *p* < 0.01), parenting behaviour (r = −0.29, *p* < 0.01), and peer attachment (r = −0.25, *p* < 0.01), while ego-resilience showed significantly positive correlations between parenting behaviour (r = 0.25, *p* < 0.01) and peer attachment (r = 0.34, *p* < 0.01); parenting behaviour was significantly positively correlated with peer attachment (r = 0.46, *p* < 0.01).

### 3.3. Mediating Effects of Ego-Resilience, Parenting Behaviour, and Peer Attachment on the Relationship Between Smartphone Dependency and Aggression

Table 4 shows the mediating effects of ego-resilience, parenting behaviour, and peer attachment on the relationship between smartphone dependency and aggression. We first analysed the mediating effect of ego-resilience. In the first step, we analysed the effect of the independent variable (smartphone dependency) on the dependent variable (aggression). This confirmed that smartphone dependency had a significant effect on aggression (β = 0.27, *p* < 0.001). 

In step two, we analysed the effect of the independent variable (smartphone dependency) on the mediating variable (ego-resilience). As a result, smartphone dependency showed significant results (β = −0.23 *p* < 0.001) on ego-resilience. In the third step, the independent variable (smartphone dependency) and mediating variable (ego-resilience) were simultaneously analysed to validate their influence on the dependent variable (aggression). As a result, both the independent variable (β = 0.22, *p* < 0.001) and mediating variable (β = − 0.17, *p* < 0.001) had significant effects. However, the effect of the independent variable (β = 0.22, *p* < 0.001) was less than that of the dependent variable (with the exception of the mediating variables (β = 0.27, *p* < 0.001)). Therefore, ego-resilience (a mediating variable) was found to partially mediate the relationship between smartphone dependency and aggression. As such, Hypothesis 1 was supported.

Second, we analysed the mediating effect of parenting behaviour. In step two, we analysed the effect of an independent variable (smartphone dependency) on a mediating variable (parenting behaviour). As a result, smartphone dependency showed significant results for parenting behaviour (β = −0.25, *p* < 0.001). In the third step, the independent variable (smartphone dependency) and mediating variable (parenting behaviour) were simultaneously analysed to verify their influence on a dependent variable (aggression). As a result, both the independent variable (β = 0.21, *p* < 0.001) and mediating variable (β = −0.23, *p* < 0.001) had significant effects. However, the effect of the independent variable (β = 0.21, *p* < 0.001) was reduced in relation to that of the dependent variable (β = 0.27, *p* < 0.001) (with the exception of the mediating variable (β = 0.27, *p* < 0.001)). Therefore, parenting behaviour (a mediating variable) was found to partially mediate the relationship between smartphone dependency and aggression. As such, Hypothesis 2 was supported.

Third, we analysed the mediating effect of peer attachment. In the third step, we analysed the effect of an independent variable (smartphone dependency) on a mediating variable (peer attachment). As a result, smartphone dependency showed significant results for peer attachment (β = −0.12, *p* < 0.001). In step three, an independent variable (smartphone dependency) and mediating variable (parenting behaviour) were simultaneously analysed to verify their influence on a dependent variable (aggression). As a result, both the independent variable (β = 0.24, *p* < 0.001) and mediating variable (β = −0.22, *p* < 0.001) had significant effects. However, the effect of the independent variable (β = 0.24, *p* < 0.001) was reduced in relation to that of the dependent variable (β = 0.27, *p* < 0.001) (with the exception of the mediating variable). Therefore, peer attachment (a mediating variable) was found to partially mediate the relationship between smartphone dependency and aggression. As such, Hypothesis 3 was supported. Table 5 shows the direct and indirect effects of these variables.

We conducted bootstrapping using the PROCESS macro to verify the mediating effects (Table 6). A total of 10,000 samples were re-extracted for the bootstrapping process; the lower limit (LLCI) and upper limit (ULCI) of the median effect factor (estimated at a 95% confidence interval) were thus obtained. As a result of the mediating effect test, ego resilience between smartphone dependency and aggression and parenting attitude and peer attachment did not include 0 between the lower limit value and the upper limit value of the confidence level. This was statistically significant. 

### 3.4. Moderating Effects of Ego-resilience, Parenting Behaviour, and Peer Attachment on the Relationship Between Smartphone Dependency and Aggression

Table 7 shows the moderating effects of ego-resilience, parenting behaviour, and peer attachment on the relationship between smartphone dependency and aggression. We first analysed the moderating effect of ego-resilience.

In step 3, the independent variables, moderating variables, and interaction terms were all injected. Here, smartphone dependency (β = 0.23, *p* < 0.001), ego-resilience (β = −0.19, *p* < 0.001), and interaction terms (β = −0.05, *p* < 0.05) were shown to significantly predict aggression (the explanatory power of the model was 10%). As such, Hypothesis 4 was supported. Ego-resilience had both significant mediating and moderating effects. These findings supported previous studies indicating that self-elasticity had a significant impact on smartphone addiction [38]. Other studies have also indicated that self-elasticity is a positive variable affecting the improvement of adolescent mental adaptability [39]. As such, Hypothesis 4 was supported.

Second, we analysed the moderating effect of parenting behaviour. This analysis showed that step one (β = 0.27, *p* < 0.001) and step two (β = −0.23, *p* < 0.001) had significant impacts on aggression. As such, Hypothesis 5 was supported. This is consistent with Kim’s [40] suggestion that problematic childhood behaviours are less likely to occur when families (particularly parents) communicate positively. That is, the more positive behaviours that are exhibited, the lower aggression is among children. On the other hand, more negative parenting behaviours are associated with higher aggression among children [18]. In step three we looked at the impact of both the independent and dependent variables and interaction terms on aggression. However, none were statistically significant. In other words, parenting behaviour was shown to affect aggression, but no clear moderating effects were detected for parenting behaviour on the relationship between smartphone dependency and aggression. It can thus be inferred that the impact of parenting behaviour may be reduced because smartphone dependency has a greater impact on aggression. As such, Hypothesis 5 wasn’t supported.

Third, we analysed the moderating effect of peer attachment. This analysis showed that step one (β = 0.27, *p* < 0.001) and step two (β = −0.22, *p* < 0.001) had significant impacts on aggression. As such, Hypothesis 6 was supported. Low peer attachment (meaning that individuals were not supported by their peers) resulted in increased aggressiveness [29]. A previous study showed that higher peer attachment was associated with greater risks of both mobile phone addiction and juvenile delinquency [25]. While we looked at the impact of both the independent and dependent variables and interaction terms on aggression in step three, none were statistically significant. It can thus be inferred that the impact of peer attachment may have been somewhat reduced because smartphone dependency had a greater impact on aggression. As such, Hypothesis 6 wasn’t supported.

## 4. Discussion

Smartphones are an indispensable tool for young people who are growing up in a digital environment. The ability to use smart devices properly is essential for adolescents, as technological advancements have changed the skills that are now needed. Adolescents find and utilise the information they need using various applications on their smartphones, as well as easily and conveniently express themselves and communicate with their friends [4]. For example, it utilises various functions such as alarm functions, map searches, calendar management, data searches, credit card payments, banking, mobile ID cards, cameras, lectures, and learning tools. On the other hand, digital media can be addictive, and is especially so at a young age [41]. The overuse of smartphones can cause more problems for young people. Thus, the careful use of smartphones is necessary because misuse can lead to behavioural problems such as overuse and aggression due to the characteristics of adolescents who lack self-control [42].

This study identified the factors that mediated and moderated the relationship between smartphone dependency and aggression among middle school students and which thereby fostered psychological growth. This analysis was conducted among 1863 smartphone users based on data collected by the 7th Korean Children and Youth Panel Survey (KCYPS), which was conducted by Korea’s National Youth Policy Institute [2]. Middle school students’ thoughts and behaviours did not suddenly manifest over the course of one year, but are linked from the birth of the student to their present-day life experience, and are cognitively, emotionally, and behaviourally built up within them. In particular, the variables of this study, namely, aggression, self-resilience, parenting behaviour, and peer attachment, are formed through 12 to 14 years of experience, which is latent in the students’ internalised thoughts and emotional behaviour. Several meaningful results were obtained.

First, ego-resilience had partial mediating and moderating effects on the relationship between smartphone dependency and aggression. Previous studies have shown that higher smartphone dependency is associated with lower ego-resilience among elementary school students [16,43]. One study found that low ego-resilience among middle school students had a negative emotional effect [44]. However, this study found that ego-resilience is a mediator between smartphone dependency and aggression. Ego-resilience also directly affected aggression one year after the occurrence of an event. This supported the findings of a previous study showing that high ego-resilient adolescents experienced both less aggression and fewer externalisation problems [45]. This study further showed that ego-resilience had a moderating effect on the relationship between smartphone dependency and aggression. This is consistent with previous studies indicating that ego-resilience can control smartphone use among high school students [46]. It is also in line with Jung’s [20] study showing that the relationship between risk factors and problematic behaviours among adolescents was at least partially controlled by ego-resilience, which mitigated risk factors and lowered the possibility of problematic behaviours. Lee [24] reported that ego-resilience had a significant influence on reducing problematic behaviours among male delinquent adolescents. These results suggest that ego-resilience can mediate and moderate smartphone dependency among adolescents, thereby preventing aggressive behaviours. 

Second, parenting behaviour partially mediated the relationship between smartphone dependency and aggression. However, it did not have a moderating effect. There is a link between low internet usage and free and open parent-child communication [46]. Cho and Lee [22,47] also showed that positive parenting behaviour reduced internalisation and externalisation problems in adolescents. Previous studies on middle school students further showed that internet addiction and positive parenting behaviours were negatively correlated [48,49]. Finally, a correlation analysis between mobile phone dependency and parenting behaviour showed that mobile phone dependency was positively associated with negative parenting behaviour, but was negatively associated with positive parenting behaviour [42,50]. These results are in line with this study’s findings. However, parenting behaviour was not a significant moderating variable in the relationship between smartphone dependency and aggression. 

Third, peer attachment partially mediated the relationship between smartphone dependency and aggression, but there was no moderating effect. This is in line with a study suggesting that smartphone overuse lowers the quality of peer relationships [41,51]. Lee [52] reported that higher peer attachment was associated with decreased delinquent behaviour. Further, it was reported that better peer-to-peer relationships resulted in increased well-being and were effective in reducing aggressive behaviour [53,54]. However, peer attachment was not a significant moderating variable in the relationship between smartphone dependency and aggression. This finding is in line with a study by So [55], which reported that the effect of internet addiction on aggression was not controlled by peer attachment. As such, there is a need to develop and apply interventions involving individuals, households, and schools in order to prevent and control smartphone overuse among middle school students. 

This study’s limitations and suggestions for future research are as follows. First, this was a cross-sectional study, which precludes any causal inferences. However, more rigorous research can be accomplished through a longitudinal study using different timeframes. Second, only some of the scale items were used in the KCYPS. There may thus be limitations in reflecting findings using the overall scale. Further studies should use all scale items. Third, this study used ego-reporting questionnaires. Thus, its findings may be affected by personal factors (e.g., question interpretation skills and subjective errors). Future research should use a variety of research methods.

## 5. Conclusions

This study’s findings contribute to existing research on adolescent health in three areas:

(1) It produced meaningful information about the variables moderating and mediating the relationship between smartphone dependency and aggression among middle school students. This issue was comprehensively examined based on individual, household, and societal variables. One of the goals of nursing is to consider humans as an integrated whole and to help them maintain and optimise their health while interacting with their environment. Thus, ego-resilience (personal), parenting behaviour (family), and peer attachment (society) were used as variables to understand their interactions with both the internal and external environments from biological, psychological, and social perspectives. This study specifically examined whether personal capabilities, support from parents, and support from friends created a synergistic effect.
(2) Ego-resilience controlled the impact of smartphone dependency on aggression, while smartphone dependency mediated this relationship with aggression. These findings suggest that the development of ‘ego-resilience’ is important for reducing the side effects of smartphone overuse among both adolescents and young adults. As such, adolescent aggression can be prevented through education designed to increase ego-resilience. Higher levels of both family and peer support also increase ego-resilience and positive self-concepts among adolescents [53]. A previous study also showed that social support could enhance ego-resilience [55], thus suggesting the effectiveness of creating an environment that provides sufficient social support.
(3) Middle school students who are highly dependent on smartphones require a variety of programs and activities designed to improve both parenting behaviour and peer relationships. This will reduce the impact of smartphone dependency on aggression by moderating parenting behaviour and peer attachment. Parents should exhibit consistent behaviour, provide affection and attention, and rationally explain instances of child misbehaviour to prevent aggression that results from smartphone dependence. Such parenting behaviours positively affect the development of adolescents, reduce their aggressive tendencies, and foster healthy growth. It is thus necessary to provide education, counselling, and various programs for adolescents who are exposed to smartphones in order to control their aggressive behaviours and to help them develop social and communication skills.

## Figures and Tables

**Table 1:** Demographic information.

Measures		Sex		Total
		Male	Female	
Age	12 (Born in 2004)	25	26	51 (2.7%)
	13 (Born in 2003)	898	901	1799 (96%)
	14 (Born in 2002)	8	6	14 (1.3%)
	Total	916 (49.2%)	947 (50.8%)	1863 (100%)
Grade		Middle School Freshman		1863 (100%)

**Table 2:** Descriptive statistics on research variables (*N* = 1863).

Variable	Score Range	Min	Max	M	SD
Smartphone dependency	7–28	7	28	17.26	4.35
Aggression	6–24	6	22	10.78	3.13
Ego-resilience	14–56	21	56	39.80	5.41
Parenting behaviour	21–84	33	84	61.21	8.18
Peer attachment	9–36	14	36	28.28	3.77

**Table 3:** Correlations between research variables.

Variable	Smartphone Dependency	Aggression	Ego-Resilience	Parenting Behaviour	Peer Attachment
Smartphone dependency	1				
Aggression	0.27 **	1			
Ego-resilience	−0.27 **	−0.23 **	1		
Parenting behaviour	−0.25 **	−0.29 **	0.25 **	1	
Peer attachment	−0.12 **	−0.25 **	0.34 **	0.46 **	1

** *p* < 0.01.

**Table 4:** Mediating effects of ego-resilience, parenting behaviour, and peer attachment on the relationship between smartphone dependency and aggression.

Step	Pathway	B	S.E	β	t	*R*²	*F*
1	Smartphone dependency → Aggression	0.19	0.02	0.27	11.9 ***	0.27	141.79 ***
2	Smartphone dependency → Ego-resilience	−0.28	0.03	−0.23	−10.00 ***	0.051	100.06 ***
3	Smartphone dependency → Aggression	0.16	0.02	0.24	10.00 ***	0.10	104.51 ***
	Ego-resilience → Aggression	−0.10	0.01	−0.17	−7.91 ***		
1	Smartphone dependency → Aggression	0.19	0.02	0.27	11.9 ***	0.27	141.79 ***
2	Smartphone dependency → Parenting behaviour	−0.47	0.04	−0.25	−11.02 ***	0.061	121.48 ***
3	Smartphone dependency → Aggression	0.15	0.02	0.21	9.29 ***	0.12	129.21 ***
	Parenting behaviour → Aggression	−0.09	0.01	−0.23	−10.41 **		
1	Smartphone dependency → Aggression	0.19	0.02	0.27	11.9 ***	0.27	141.79 ***
2	Smartphone dependency → Peer attachment	−0.10	0.02	−0.12	−5.11 ***	0.01	26.10 ***
3	Smartphone dependency → Aggression	0.17	0.02	0.24	10.97 ***	0.12	123.62 ***
	Peer attachment → Aggression	−0.18	0.02	−0.22	−9.90 ***		

** p < 0.01; *** p < 0.001.

**Table 5:** Direct and indirect effects of variables (standardisation factor).

Pathway	Direct Effect	Indirect Effect	Total Effect
Smartphone dependency → Ego-resilience	−0.23 ***	-	−0.23 ***
Ego-resilience → Aggression	−0.17 ***	-	−0.17 ***
Smartphone dependency → Aggression	0.27 ***	0.04 ***	0.31 ***
Smartphone dependency → Parenting behaviour	−0.25 ***	-	−0.25 ***
Parenting behaviour → Aggression	−0.23 ***	-	−0.23 ***
Smartphone dependency → Aggression	0.27 ***	0.06 ***	−0.33 ***
Smartphone dependency → Peer attachment	−0.12 ***	-	−0.12 ***
Peer attachment → Aggression	−0.22 ***	-	−0.22 ***
Smartphone dependency → Aggression	0.27 ***	0.03 ***	0.30 ***

*** *p* < 0.001.

**Table 6:** Verifying the bootstrapping mediation effect.

Path	Indirect Effect	Boot S.E	95%	
			Boot LLCI	Boot ULCI
Smartphone dependency → Ego-resilience → Aggression	0.029	0.0052	0.0195	0.0399
Smartphone dependency → Parenting Behaviour → Aggression	0.042	0.0059	0.0304	0.0538
Smartphone dependency → Peer attachment → Aggression	0.018	0.0045	0.0101	0.0275

LLCI = lower limit. ULCI = upper limit.

**Table 7:** Moderating effects of ego-resilience, parenting behaviour, and peer attachment on the relationship between smartphone dependency and aggression.

Variable		Step 1				Step 2				Step 3			
		B	S.E	β	*t*	B	S.E	β	*t*	B	S.E	β	*t*
	Smartphone dependency	0.19	0.02	0.27	11.90 ***	0.16	0.02	0.27	10.00 ***	0.16	0.02	0.23	10.11 ***
	Ego-resilience	-				−0.10	0.01	−0.18	−7.90 ***	−0.11	−0.01	−0.19	−8.14 **
	Smartphone dependency × Ego-resilience	-				-				−0.01	0.00	−0.05	−2.09 *
*R²*		0.071				0.101				0.103			
	Smartphone dependency	0.19	0.02	0.27	11.90 ***	0.15	0.02	0.21	9.28 ***	0.15	0.02	0.21	9.37 ***
	Parenting behaviour	-				−0.09	0.00	−0.23	10.41 ***	−0.09	0.01	−0.24	10.51 ***
	Smartphone dependency × Parenting behaviour					-	−0.00	0.00	−0.03	−1.45			
*R²*		0.071				0.122				0.147			
	Smartphone dependency	0.19	0.02	0.27	11.90 ***	0.17	0.02	0.24	10.96 ***	0.17	0.02	0.24	10.74 ***
	Peer attachment	-				−0.18	0.02	−0.22	−9.90 ***	−0.18	0.02	−0.22	−9.87 ***
	Smartphone dependency × Peer attachment	-				-				−0.00	0.00	−0.01	0.56
*R²*		0.071				0.117				0.117			

*** p < 0.001, ** *p*< 0.01, * *p* < 0.05.

## Appendix A

This is the questionnaire used in this study. We extracted and translated the questions from the 7th Korean Children’s Youth Panel Survey (KCYPS). The contents of the questionnaire were as follows:

*1. Do You Have a Cell Phone?*

(1) I have a cell phone; (2) I don’t have a cell phone

*2. If You Have a Cell Phone, What Kind of Cell Phone Do You Have?*

(1) Smartphone; (2) Feature phone; (3) I don’t know what kind it is

*3. Smartphone Dependency*

This is a question about what you think of your phone. Please answer the items that apply to you for each of the following questions (over the past year).

**Questions**	**Strongly Agree**	**Agree**	**Disagree**	**Strongly Disagree**
1. I spend more and more time using my cell phone. (reverse-coded)	1	2	3	4
2. I have felt anxious over the past year when not taking a cell phone with me. (reverse-coded)	1	2	3	4
3. I’m anxious if no one contacts with my cell phone. (reverse-coded)	1	2	3	4
4. When I use my cell phone time passes quickly. (reverse-coded)	1	2	3	4
5. I have felt isolated when I am without a cell phone. (reverse-coded)	1	2	3	4
6. Without a cell phone, I can’t live because it’s uncomfortable. (reverse-coded)	1	2	3	4

*4. Aggression*

This is a question about your usual behaviour. Please answer the items that apply to you for each of the following questions (over the past year).

**Questions**	**Strongly Agree**	**Agree**	**Disagree**	**Strongly Disagree**
1. Even small mistakes made by others have easily upset me over the past year. (reverse-coded)	1	2	3	4
2. I have wanted to interfere with what someone else was doing over the past year. (reverse-coded)	1	2	3	4
3. If someone won’t let me do what I want, I’ll fight with them. (reverse-coded)	1	2	3	4
4. I’ve been fighting about minor issues. (reverse-coded)	1	2	3	4
5. Sometimes I am angry all day. (reverse-coded)	1	2	3	4
6. I sometimes cry for no reason. (reverse-coded)	1	2	3	4

*5. Ego-Resilience*

This is a question about what you think of yourself. Please answer the items that apply to you for each of the following questions (over the past year).

**Questions**	**Strongly Agree**	**Agree**	**Disagree**	**Strongly Disagree**
1. I have been generous with my friends. (reverse-coded)	1	2	3	4
2. Even when unexpected things happen, I have been able to get over them. (reverse-coded)	1	2	3	4
3. I like to try new things that I don’t usually do well. (reverse-coded)	1	2	3	4
4. I make a good impression on people. (reverse-coded)	1	2	3	4
5. I enjoy eating new kinds of food. (reverse-coded)	1	2	3	4
6. I am a very energetic person. (reverse-coded)	1	2	3	4
7. When I go to the same place, I like to go a different way. (reverse-coded)	1	2	3	4
8. I am more curious than others. (reverse-coded)	1	2	3	4
9. I usually think a lot before acting. (reverse-coded)	1	2	3	4
10. I like to do new and different kinds of work. (reverse-coded)	1	2	3	4
11. My life is full of interesting things every day.(reverse-coded)	1	2	3	4
12. I can say with confidence that I am a strong-willed person. (reverse-coded)	1	2	3	4
13. Sometimes others upset me, but I can reliably manage my mood. (reverse-coded)	1	2	3	4
14. Most of the people I meet are good people. (reverse-coded)	1	2	3	4

*6. Parenting Behaviour*

This is a question about what you think of your parents (or guardians if you do not have parents). Please answer the items that apply to you for each of the following questions (over the past year).

**Category**	**My Parents Are~**	**Strongly Agree**	**Agree**	**Disagree**	**Strongly Disagree**
Monitoring	1. My parents know where I’m going after school. (reverse-coded)	1	2	3	4
	2. My parents know how I spend my time. (reverse-coded)	1	2	3	4
	3. If I go out, my parents know when I will come home. (reverse-coded)	1	2	3	4
Affection	4. My parents respect my opinion. (reverse-coded)	1	2	3	4
	5. My parents often express that they like me. (reverse-coded)	1	2	3	4
	6. My parents give me courage when I am frustrated. (reverse-coded)	1	2	3	4
	7. My parents often compliment me. (reverse-coded)	1	2	3	4
Reasoning	8. They explain their reasoning to me (protective) rather than making me blindly and unconditionally follow their decisions. (reverse-coded)	1	2	3	4
	9. They explain why something is a bad behaviour before I get scolded when I do something bad. (reverse-coded)	1	2	3	4
	10. If I make unreasonable demands, they explain why it doesn’t work. (reverse-coded)	1	2	3	4
Over-expectation	11. My parents are nervous about the things my peers are doing that I don’t do.	1	2	3	4
	12. My parents always emphasise that when I do something I should win.	1	2	3	4
	13. My parents intervene in small things.	1	2	3	4
	14. My parents often force me not to do what I want.	1	2	3	4
Inconsistency	15. The expectations of my parents (protective) are always greater than my ability and it is burdensome.	1	2	3	4
	16. I wish my parents were less concerned about me.	1	2	3	4
	17. I emphasise that I have to be better than others in every way.	1	2	3	4
	18. My parents force me to study more than anything else.	1	2	3	4
Intrusiveness	19. This varies according to the moods of my parents, kind or not.	1	2	3	4
	20. Sometimes if I make the same mistakes my parents are angry, and sometimes they are not.	1	2	3	4
	21. When a guest comes over or we go out, my parents (protective) have a different attitude toward me.	1	2	3	4

*7. Peer Attachment*

This is a question about what you think of your friends. Please answer the items that apply to you for each of the following questions (over the past year).

**Questions**	**Strongly Agree**	**Agree**	**Disagree**	**Strongly Disagree**
1. My friends respect my ideas during our conversations. (reverse-coded)	1	2	3	4
2. My friends listen to what I say. (reverse-coded)	1	2	3	4
3. I talk to my friends about my troubles and problems. (reverse-coded)	1	2	3	4
4. My friends understand me well. (reverse-coded)	1	2	3	4
5. I can tell my heart to my friends. (reverse-coded)	1	2	3	4
6. I believe in my friends. (reverse-coded)	1	2	3	4
7. I want to make other friends instead of my friends.	1	2	3	4
8. I have felt alone or lonely even when I have been with my friends over the past year.	1	2	3	4
9. My friends don’t know how I’m doing these days.	1	2	3	4
